# Service providers’ adaptations to facilitate family-centered care for children with cerebral palsy: a qualitative study

**DOI:** 10.1186/s12875-026-03251-3

**Published:** 2026-03-24

**Authors:** Silje Karin Selfors Sjøseth, Veslemøy Guise, Karina Aase, Maren Kristine Raknes Sogstad

**Affiliations:** 1 SHARE – Centre for Resilience in Healthcare, Faculty of Health Sciences, University of Stavanger, Stavanger, Norway; 2 Centre for Care Research, NTNU Gjøvik, Gjøvik, Norway

**Keywords:** Cerebral palsy (CP), Children and families care needs, Service providers, Adaptations, Family-centered care (FCC)

## Abstract

**Background:**

Family-centered care (FCC) establishes the standard for service provision for children with cerebral palsy (CP) through several integrative measures. However, to achieve true family-centeredness, long-term services must remain flexible and responsive to evolving situations and contexts throughout childhood, requiring continuous adaptations by service providers. Despite this need, research examining how practitioners adapt their practices to deliver effective services for children and their families is limited. Therefore, this study aims to explore the adaptations employed by service providers to facilitate family-centered care for children with cerebral palsy.

**Methods:**

Data were collected through focus groups with service providers and observations of multidisciplinary coordination meetings.

**Results:**

Service providers consistently adapted their practices to address the care needs of children with CP and their families. These adaptations were considered essential due to the wide variation in clinical outcomes associated with CP, the presence of comorbidities and additional diagnoses, and the diverse and evolving needs of families. Through these adaptations, service providers supported families by creating trusting relationships, ensuring early interventions, delivering individualized care, and negotiating differing perspectives. They also organized care by promoting collaboration among stakeholders and managing information effectively.

**Conclusion:**

By adapting their practices to emerging situations and needs, service providers demonstrated situational awareness and implemented flexible approaches to FCC aligned with families’ evolving requirements. These adaptations helped compensate for systemic shortcomings, ensuring coordinated and integrated care consistent with a family-centered approach.

**Supplementary Information:**

The online version contains supplementary material available at 10.1186/s12875-026-03251-3.

## Background

Children with cerebral palsy (CP) and their families require a wide range of services throughout childhood, necessitating a multidisciplinary and holistic approach [1–6]. These services involve multiple providers across healthcare, education, social services, and family support, each with distinct responsibilities and relationships to children and families. Given the complexity of CP, the diversity of family needs, and the intricacies of service systems [7, 8], the provision of long-term care must remain adaptable to specific situations and contexts to effectively uphold the principles of family-centered care (FCC) [9–13].

FCC serves as the guiding standard for service delivery to children with CP, recognizing parents as co-providers, co-decision-makers, and co-facilitators in their child’s care [14]. This partnership is grounded in core concepts such as respect, dignity, information sharing, participation, and collaboration. FFC emphasizes the strengths of the child and family rather than their limitations [15]. It promotes family autonomy by enabling informed choice and control, while fostering a therapeutic environment that supports collaborative relationships between families and service providers [15, 16].

Moreover, FCC services must be well-coordinated and integrated to ensure continuity of care [17]. Service integration encompasses vertical coordination between primary and secondary care, horizontal collaboration across health, education, and social services, and longitudinal continuity throughout the lifespan [18, 19]. Adaptations are critical for managing system complexity and addressing variations in patients’ care needs [20, 21]. As a core component of FCC, adaptations requires healthcare providers to tailor care delivery to the unique needs, goals, preferences, and contexts of each family [11].

Increasing specialization of services, combined with complex diagnoses and multifaceted care requirements, demand that service providers implement both individual and collective adaptations to maintain high-quality care [20, 22, 23]. These adaptations enables complex systems to self-organize, navigate conflicting goals, reassess priorities, innovate, and respond to emerging external demands [24]. Furthermore, such adaptations are informed by the competence and experience of service providers’, encompassing their knowledge, skills, attitudes, and situational awareness [12, 25].

Effective implementation of FCC through adaptive practices requires a careful balance between empowering parents and providing appropriate support. This balance fosters engagement and autonomy without introducing additional stress or unwanted responsibility [15]. Achieving this balance depends on continuous adaptation to families’ evolving needs. However, research on how these adaptations are operationalized in clinical practice remains limited, creating gaps in understanding how FCC principles are applied in everyday service delivery [15]. Furthermore, the lack of conceptual clarity and limited insight into how FCC evolves over time contribute to its inconsistent implementation [15, 26]. Guided by the theoretical framework of adaptations and FCC, this study aims to explore how providers adapt their practices to facilitate FCC for children with CP. This article focuses on how service providers adapt in their interactions with families, while the families’ perspectives are addressed in a subsequent article by the same authors [27]. The following research question is proposed for this article:

- How do service providers enact adaptations to facilitate family-centered care for children with cerebral palsy?

## Methods

### Study design

A qualitative design incorporating focus groups and observations was employed to explore how service providers enact adaptations to facilitate FCC to children with CP [28–30]. In this study, service providers included professionals from various care units who offer services to children and their families throughout long-term care.

### Context

In Norway, services for children with CP and their families are delivered through municipal, regional, and governmental units organized across health and care services, labor and social services, educational services, and child and family services [31, 32]. These services include assessments and treatment within specialist healthcare, followed by long-term services in primary healthcare settings [33–35]. Children also receive social and educational support through the pedagogical-psychological service, which provides assistance in kindergarten and school as needed [36–39]. In addition, financial and social support services are available to families based on their specific challenges and needs [40, 41], along with family counseling services for those experiencing challenging care situations [42, 43]. To ensure high quality care, long-term care guidelines have been established within specialist healthcare, with most services delivered through regional habilitation units [44]. Coordination of services is supported by the child’s legal right to an assigned coordinator from primary healthcare [45, 46]. Consequently, habilitation services for children with CP represent a shared responsibility between specialist and municipal healthcare providers [44, 46]. Personnel in the child’s kindergarten and school are responsible for implementing agreed pedagogical measures and participating in regular multidisciplinary coordination meetings [36, 37, 44, 46].

### Recruitment and data collection

In a previous study [47], six families, each with a child aged between eight and twelve years and a primary diagnosis of CP were recruited. Interviews with these families were used to identify service units important to their short-term or long-term care. In the present study, these service units were contacted and invited to participate in focus groups, resulting in the inclusion of twenty-nine service providers. The service providers were not informed of the families’ participation in the earlier study. Furthermore, although the service providers worked with children with CP within their respective municipalities and counties, they may not have been directly involved with the previously interviewed families. To ensure anonymity and broader representation, service providers were recruited based on their professional roles rather than as specific individuals. An overview of the focus group participants is presented in Table 1.

**Table 1:** Overview of focus group participants

Service provider profession or position	Services represented
Pediatrician (*N* = 1) Neuropsychologists (*N* = 6) Physiotherapists (*N* = 3) Occupational therapists (*N* = 3)	Child and Adolescent Habilitation unit at a university hospital, specialist healthcare services
Physiotherapists (*N* = 4) Occupational therapists (*N* = 4) Coordinators (*N* = 4)	Primary healthcare services
Advisors from the school section (*N* = 2) Advisors from the kindergarten section (*N* = 2)	Pedagogical Psychological Service, educational sector

Focus groups were employed to explore participants’ views and perspectives [28, 48]. In total, six focus groups were conducted, each comprising four to six participants to encourage active engagement and facilitate effective communication [49]. The groups were organized as follows: Group 1 included six neuropsychologists from the Child and Adolescent Habilitation Unit; Group 2 consisted of three physiotherapists and three occupational therapists from the same unit; Group 3 included two physiotherapists and two occupational therapists from municipality 1; Group 4 included two physiotherapists and two occupational therapists from municipality 2; Group 5 comprised two advisors from the school section and two from the kindergarten section within the Pedagogical Psychological Service; and Group 6 included two coordinators from municipality 1 and two coordinators from municipality 2. Additionally, one individual interview was conducted with a pediatrician who was unable to attend the focus group with participants from the Child and Adolescent Habilitation Unit. This interview followed the same question guide as the focus groups to ensure consistency in data collection.

Focus groups and the individual interview lasted between 108 and 148 min and were guided by an interview guide exploring how service providers collaborated with families and other professionals, how they addressed families’ care needs, and what they considered essential for fostering effective collaboration. At the end of each focus group and the individual interview, a summary of the discussion was presented to participants, allowing them to verify and supplement the information provided. All focus groups were facilitated by researcher SKSS, a trained occupational therapist with no prior relationship to participants, together with one additional researcher (VG, KA, or MKRS). The individual interview was conducted by SKSS. All focus groups and the individual interview were conducted in Norwegian, and audio recordings were transcribed verbatim in Norwegian by SKSS.

In addition, observations were carried out during multidisciplinary coordination meetings involving parents, school personnel, and service providers engaged in the child’s long-term care. Table 2 provides an overview of service providers included in the observations. Two participants in the observations had previously participated in focus groups. Observations were conducted by SKSS and documented using an observational guide with predefined questions [50]. Focus groups, the individual interview and observations were conducted in parallel. The interview and observation guides are available as supplementary files.

**Table 2:** Overview of observation participants

Service provider profession or position	Services represented
Neuropsychologists (*N* = 1) Physiotherapists (*N* = 2) Occupational therapists (*N* = 2)	Child and Adolescent Habilitation unit at a university hospital, specialist healthcare services
Physiotherapists (*N* = 3) Occupational therapists (*N* = 3) Coordinators (*N* = 4)	Primary healthcare services
Contact teachers (*N* = 4) Assistant (*N* = 3) School inspectors (*N* = 5) Health nurse (*N* = 2) The school principal (*N* = 2)	The child’s school, educational sector
Advisors (*N* = 3)	Pedagogical Psychological Service, educational sector

### Analysis

Focus groups and the individual interview served as the primary data sources for this study, while observation notes provided complementary insights that helped confirm and contextualize the interview findings. Two service providers who participated in focus groups were also participants in the observations. All service providers involved in observations held the same professional roles as those participating in the focus groups, thereby reinforcing and validating the perspectives described. To explore how service providers enact adaptations, a thematic analysis was conducted to identify, analyze, and report patterns within the data [51]. The analysis followed Braun and Clarke’s six-phase approach, beginning with transcription and concluding with the final reporting of findings [52].

- Phase One: Researcher SKSS read the transcriptions from the focus groups and the individual interview multiple times to gain familiarity with the data and begin generating initial notes. Guided by the research question, the theoretical framework of FCC and the concept of adaptation were central, making it essential to maintain awareness of these constructs throughout the analysis [51, 53]. Despite this theoretical foundation, SKSS adopted an open and inclusive approach to ensure that all aspects of the data were comprehensively considered.
- Phase Two: SKSS systematically coded the entire dataset, identifying statements that described service providers’ adaptations, the contexts in which these adaptations occurred, the factors that prompted them, and their perceived outcomes.
- Phase Three: The generated codes were organized into potential themes that captured the different types of adaptations enacted by service providers. During this phase, similar adaptations were grouped together to form coherent thematic patterns.
- Phase Four: The themes were reviewed against the coded extracts to ensure internal consistency and coherence. During this phase, overlapping subthemes were merged, and adjustments were made to refine the overall thematic structure.
- Phase Five: Each theme was evaluated in relation to the overarching narrative emerging from the data. Clear definitions and concise names were developed to capture the essence of each theme.
- Phase Six: The final themes were refined and presented to convey a coherent and compelling account of how service providers adapt their practices to support family-centered care. Participant quotes were translated and incorporated to illustrate the different subthemes.

To ensure reflexivity throughout the six analytical steps, the research team engaged in collaborative discussions regarding the codes, themes, and subthemes [53, 54]. These discussions facilitated continuous reflection on the analytical framework, the relationships between codes and themes, and the coherence of the thematic structure. The final themes were selected based on their capacity to represent a meaning-based narrative that accurately captured participants’ responses during the focus groups and the individual interview [53].

## Results

Our results document that service providers employed a range of adaptations to implement flexibility in FCC for children with CP. These adaptations were described by service providers as necessary due to several factors, including the wide variation in clinical outcomes associated with CP, the presence of co-occurring conditions and additional diagnoses, and the evolving needs of families. Through our analysis, we identified two overarching themes, each with corresponding sub-themes, that capture how service providers adapted their practices to facilitate FCC. These are presented in Table 3.

**Table 3:** Adaptations to facilitate FCC, themes, and sub-themes

Themes	Sub-themes
Adaptations to support families	Creating trusting relationships Ensuring early interventions Ensuring individualized care Negotiating divergent perspectives
Adaptations to organize care	Managing information Facilitating collaboration

### Adaptations to support families

Service providers supported families in long-term care by implementing adaptations aimed at creating trusting relationships, ensuring early interventions, ensuring individualized care, and negotiating divergent perspectives.

#### Creating trusting relationships

Service providers described creating trusting relationships, where families felt their experiences were understood and acknowledged, as a necessary first step in delivering FCC. To establish these relationships, they reported adapting their practices by spending additional time to get to know families, meeting families on their terms, providing tailored information, ensuring availability, and addressing challenges promptly.

During the child’s initial assessment period, service providers scheduled meetings aimed at gaining a deeper understanding of the family. In some cases, this involved more meetings than normally offered. In these meetings, service providers reported actively listening to families, inquiring about care needs, family relationships, including those with other children, and available resources such as support networks. Service providers also emphasized efforts to provide concise information about the CP diagnosis and available services to minimize misunderstandings, frustration, or unnecessary concern.

To maintain trusting relationships, service providers described making themselves available to families beyond standard expectations. Service providers considered this as essential due to the families ongoing need for support and reassurance throughout long-term care, which involved multiple assessment and treatment periods for their child. Service providers noted that questions and challenges frequently arose during these phases, alongside evolving care needs as explained in the following:

*“An important key is to be available*,* just to give your email address and phone number and say that they can call if things are difficult. It gives them security knowing that if things go completely wrong*,* they have someone they can contact. It does cost [me] something*,* but I have never experienced that it has been misused. When I have received phone calls it has been from really frustrated parents in difficult situations.”* (Pediatrician, specialist healthcare).

Service providers described the importance of responding promptly to families’ requests to sustain trusting relationships. Although some inquiries concerned minor issues that could be easily resolved, they noted that leaving such matters unattended could create challenges for families. For example, service providers facilitated the exchange of referrals and assessment reports with other professionals and acted as intermediaries by requesting information and follow-up services on behalf of parents. When a family’s request required more time to address, service providers explained that they provided brief status updates via email or phone calls to keep parents informed. Additionally, coordinators proactively contacted families during assessment or treatment periods for their child, or after lapse in communication, to check for concerns. These proactive measures were described as essential for maintaining availability, fostering trust, and enabling families to access services and communicate their care needs effectively.

#### Ensuring early intervention

During consultations, service providers from both specialist and primary healthcare services reported addressing the characteristics of the CP diagnosis, including its associated challenges and care needs, from a life-course perspective. These adaptations were described as integral to conversations aimed at empowering parents and promoting a proactive approach to care.

To support parents in becoming active partners in their child’s long-term care and training, service providers described adopting a gradual approach providing information and introducing aids and services. This approach aimed to ensure parental involvement in care processes. Service providers reported tailoring the information according to their perception of what parents were ready to receive and were cautious about introducing multiple measures simultaneously. In several situations, they noted challenges in identifying needs early and anticipating future care requirements. In response, service providers described adopting a proactive approach by mobilizing follow-up resources as early as possible. Examples included referrals to physiotherapy and occupational therapy units, as well as the pedagogical-psychological service, to facilitate the establishment of follow-up services in kindergartens and schools. Service providers explained that early intervention enhanced parental involvement in care services and fostered a better understanding and sense of ownership of the agreed care measures. This was described by the service providers as strengthening family resources and potentially preventing secondary challenges in the child’s development:

*“There is a big difference between families who have been involved in early intervention versus those who have caught up later. In early intervention*,* it is the parents who do 99% of the training in their own home based on our guidance. Then it becomes more integrated into their everyday life and then they carry on in a different way. Those parents are very involved. They have ownership and an understanding in a completely different way. Additionally*,* the benefits for the child’s development are very good.”* (Occupational therapist, primary healthcare).

#### Ensuring individualized care

Families of children with CP often experience additional challenges that affect both their care needs and their ability to engage with follow-up services. These challenges may include complex family dynamics, such as divorce or shared custody arrangements, as well as difficulties faced by the child that influence motivation and capacity to participate in treatment and exercise. When such circumstances arise, service providers from both specialist and primary healthcare settings reported setting aside standard protocols during consultations to address the family’s situation and perceptions of current challenges. Together with the family, they prioritize follow-up measures that best meet immediate needs. Providers emphasized that determining when to adhere to standard procedures and when to adapt practices requires professional judgment, often informed by experience. Experienced service providers were described as having a deeper understanding of family needs and greater confidence in modifying established protocols. In these cases, providers typically completed procedural checklists after the consultation or scheduled a follow-up meeting to address outstanding items, such as measuring leg posture:

“*It is meaningless to follow a protocol and use the consultation to talk about angles and adjustments of orthoses if the families can’t manage to follow it up because of an ongoing separation or if the most important challenge is the child’s problems with sleep*,* concentration*,* or lack of friends at school. Then we need to put the procedures aside to talk about those things because they are important for us to know. Of course*,* it is our responsibility to ensure that a scoliosis is not allowed to develop*,* but we also need to address those other important things.”* (Physiotherapist, specialist healthcare).

To minimize the number of professionals families needed to interact with, service providers in specialist healthcare reported taking on additional responsibilities. These included conducting extended assessments beyond their primary role, such as performing ADHD evaluations and delivering treatment for anxiety and depression when the child was already receiving care.

Adaptations were also implemented in situations where parents struggled to make treatment decisions for their child. For instance, in cases involving extensive orthopedic surgery, service providers offered decision-support interventions to help families navigate available options explained as follows:

*“Or we give [parents] the direct number of the surgeon so they can call after they have thought it through. After all*,* we know the parents well and often know when they need decision support. We have become better at taking responsibility for these decisions so that it is not pushed onto the parents.”* (Occupational therapist, specialist healthcare).

Adaptations to support families during particularly challenging periods included additional conversations with parents, either individually or together with their child, depending on the nature of the issue and the family’s preferences. For primary healthcare providers, these adaptations also involved allocating extra time during follow-up consultations after assessments and treatments provided by specialist healthcare services. Service providers described it as follows:

*“I think that*,* because we as the physiotherapist and occupational therapist are closer to the families*,* parents feel that contacting us is simple and straightforward. In that way we often become a conversation partner for families in the challenges they face*,* where we help them to understand the feedback. In that way we must always plan for extra time beyond just the child’s physical issues that we follow up on.”* (Physiotherapist, primary healthcare).

#### Negotiating divergent perspectives

All service providers reported making adaptations to address diverging perspectives on care needs between parents, between parents and service providers, and between parents and the child.

Disagreements between parents often involved differing views on the severity of the CP diagnosis, the child’s care needs, and the family’s need for assistance. For example, fathers were more likely to emphasize physical challenges, whereas mothers frequently highlighted social and cognitive difficulties. These differences were described as particularly pronounced in families experiencing strained relationships, such as divorce or shared custody. In such cases, service providers scheduled joint meetings with parents to listen to each perspective and normalize differing reactions and priorities. Providers noted that offering additional information and creating opportunities for parents to express their views helped bring parents closer together and facilitated agreement on follow-up measures. Differences in perspectives also emerged between parents and service providers. For example, some parents struggled to understand feedback from cognitive assessments. This difficulty was often attributed to limited prior experience with the identified challenges or uncertainty about the significance of the findings. To address this, service providers scheduled additional meetings that allowed parents to observe the challenges directly. In many cases, firsthand experience was essential for parents to comprehend and accept the assessment results. Service providers also described involving the extended interdisciplinary team to deliver coordinated and consistent feedback to families:

*“In situations where it is difficult to establish a shared understanding and we disagree with parents*,* it is important to use the interdisciplinary environment*,* public health nurse*,* school*,* the pedagogical-psychological service*,* and local physiotherapist and occupational therapist*,* to discuss issues and give joint feedback.”* (Neuropsychologist, specialist healthcare).

Service providers also described situations where differing perspectives arose between children and their parents. In these cases, providers first engaged with the child to explore possible approaches for addressing the issue. They then sought the child’s permission to share their perspective with the parents. In most instances, the child agreed, and parents expressed appreciation for having their child’s views communicated by the service providers. These conversations sometimes included encouraging parents to speak positively about the use of assistive devices and allowing the child to decide when to use equipment, such as their wheelchair.

### Adaptations to organize care

All service providers described how they worked to organize the care according to family needs. This work included adaptations to manage information and to facilitate collaboration across the multiple providers involved in services.

#### Managing information

During their work with families, service providers reported frequent challenges in sharing information that was not automatically distributed among other involved professionals. In several cases, this information was described as both difficult to obtain and deliver. Barriers to information exchange were noted between care units within specialist healthcare, between specialist and primary healthcare services, and between the healthcare and educational sectors. These difficulties were attributed to the use of different patient record systems across organizations, limited access to shared systems, and a lack of awareness regarding the information needs of other service providers. To ensure that essential information, such as assessment results, planned treatments, scheduled surgeries, changes in the child’s condition, and emerging care needs, was communicated, service providers often relied on parents as intermediaries. This included asking parents to bring reports from previous consultations, contacting them via email or phone for updates, or requesting signed consent forms to enable information sharing between providers. Additionally, service providers described discussing these challenges with one another to identify strategies for compensating for system limitations and facilitating effective information exchange:

*“To receive important information*,* we need to go outside the system*,* and it’s always a bit difficult when you call or send an email with questions about an operation for a child. But every child has a patient code that we can give so that we know we are talking about the right child to be able share information.”* (Physiotherapist, specialist healthcare).

In cases where information transfer was delayed or incomplete, such as planned hospital treatments requiring preparation and follow-up by primary healthcare providers, service providers contacted each other directly to improve coordination. This approach enabled them to reschedule treatments and ensure appropriate resource allocation, preparation, and follow-up activities described in the following:

*“After all*,* we are sometimes forgotten in the planning of Botox treatment or in case of Intrathecal baclofen therapy (baclofen pump). A lot of valuable information and planning is then lost. But we try as best we can to change the plans to ensure that preparations and follow-up is planned*,* and necessary resources are allocated.”* (Physiotherapist, primary healthcare).

To ensure coordination of services and effective information sharing among providers, all families in this study had a designated municipal coordinator who organized multidisciplinary coordination meetings. However, coordinators emphasized that extensive preparation was essential to address care needs, clarify responsibilities, and resolve disagreements, factors considered crucial for fostering a collaborative and productive atmosphere:

*“I try to talk to the parents ahead of meetings. And if there are things that they would like to have in place*,* I take a round with all the actors ahead of the meeting and try to get clarification and suggest approaches so that they can present a solution in the meeting instead of the meeting turning into a discussion. So*,* the family receives feedback in the meeting instead of the challenges being thrown back and forth between providers regarding who is responsible for the various things. I think that successful meetings depend on good preparations and that you first find the challenges the child and parents face.”* (Coordinator, primary healthcare).

#### Facilitating collaboration

Collaboration across service providers was described as particularly challenging when follow-up responsibilities were unclear. A lack of clarity regarding roles and appropriate approaches to supporting children with complex care needs often led to differing views on suitable interventions and highlighted limitations in available resources and services. Service providers reported instances where specialist healthcare teams requested follow-up from primary healthcare providers, but these requests were difficult to fulfill due to service unavailability. Additionally, primary healthcare providers noted the need to conduct their own assessments of the child and family’s care needs, following their established procedures, alongside those already performed in specialist healthcare.

To promote a consistent approach to long-term care, service providers established regular meeting forums across sectors and levels of care to collaborate at both individual and system levels. One group of providers, who experienced interdependencies in their follow-up of families, organized monthly collaboration meetings to share information about their respective services and discuss anonymized cases to clarify responsibilities and enhance coordination. With the family’s consent, specific cases were also addressed to determine which care unit had the necessary personnel and expertise to meet the family’s needs. Furthermore, service providers from both specialist and primary healthcare sought permission from families to participate in joint consultations, aiming to address service interdependencies and support a holistic approach to follow-up care.

Despite these efforts, disagreements regarding follow-up approaches still occurred. In such cases, service providers began by clarifying their respective roles, expectations, and responsibilities in the family’s long-term care to foster mutual understanding. They then provided information tailored to the situation, acknowledging that each provider’s knowledge and experience shaped their perspective. During these discussions, service providers explicitly addressed both their requests to other professionals and their own contributions to the child’s care:

*“When it comes to situations where collaboration is difficult*,* we talk together to clarify the responsibilities and set very clear and concrete objectives that everyone works toward. That the various providers’ contributions are expressed in such a way that it is measurable if it is done or not*,* what the results were*,* and if not done*,* why not?” (*Occupational therapist, primary healthcare).

Service providers described establishing a shared understanding as an ongoing process that required repeated conversations about specific challenges and potential solutions across multiple meetings. Neuropsychologists and advisors from the pedagogical-psychological service reported scheduling follow-up meetings at set intervals after initial consultations to share assessment results with both parents and the school:

*“Sometimes things need to be experienced before it is understood*,* unfortunately. Therefore*,* the second meeting is often more useful when they have gained some experiences that they can relate our assessments to. That’s why I ask to be invited to a coordination meeting after a year and I tell them to call if they experience any of the challenges that I have informed them about.”* (Advisor, the pedagogical-psychological service).

## Discussion

This study demonstrates that service providers implemented a range of adaptations within their service delivery to facilitate FCC for children with CP. Based on the findings, these adaptations can be categorized into two types: those aimed at supporting families and those focused on organizing care. The former can be described as frontstage adaptations, directed toward families, while the latter represent backstage adaptations, occurring among service providers to align and coordinate services.

The concept of social interaction within both frontstage and backstage settings has been used to explain how interactions unfold across contexts and how they are shaped by the individuals involved [55, 56]. From this perspective, adaptations by service providers can be viewed as performances tailored to different actors and situations. The backstage perspective emphasizes how providers prepare and organize care, whereas the frontstage perspective illustrates their direct engagement with families of children with CP. While adaptations are central to the implementation of FCC, it is essential to examine how service providers respond to families’ changing needs to understand how FCC evolves through these interactions. Gaining insight into how FCC is understood and practiced by service providers is crucial for addressing the diverse and evolving needs of families.

### Backstage adaptations

Service providers implemented several backstage adaptations to organize and tailor care in response to families’ needs. By managing information and facilitating collaboration, they aimed to connect interdependent follow-up services, thereby enhancing service quality [57–60]. These adaptations also ensured that services were aligned with the needs of children and families, contributing to vertical, horizontal, and longitudinal integration across care systems [18, 19].

Moreover, effective collaboration does not occur spontaneously, even when long-term care guidelines incorporate various integrative measures [34–39, 44, 45]. Service providers’ adaptations can be understood as invisible organizing work, tasks not explicitly outlined in guidelines but essential for aligning services across time and space for patients [57]. This invisible work plays a critical role in service quality, operating at a level of importance comparable to direct interactions with children and their families. Within this context, successful collaboration depends on coordinators actively sharing information among participants and carefully preparing and organizing multidisciplinary meetings.

Service providers’ adaptations to manage information highlight their critical role in ensuring the quality of follow-up care. These efforts help integrate different measures, reducing the risk of misunderstandings and potentially inappropriate treatment. However, preventing information gaps requires investment in systems and routines that enable providers to access relevant information seamlessly throughout all stages of a child and family’s long-term care [61]. Furthermore, implementing integrated care principles within regulatory and legal frameworks is essential to support information sharing across providers. This includes establishing clear guidelines and developing infrastructure that facilitates coordination and interoperability between services [62].

The adaptations enacted by service providers to align follow-up activities were influenced by differing professional perspectives, knowledge bases, and approaches. These differences reflect the varied interpretations of integrated care across providers and settings, underscoring that the concept does not carry a uniform meaning in practice [63]. To address these variations, service providers reported making adaptations during collaborative meetings to clarify roles, expectations, and perspectives. These efforts included maintaining frequent communication, setting clear goals for joint contributions to family services, and explicitly articulating both their own responsibilities and their requests to other providers [64–66].

### Frontstage adaptations

Frontstage adaptations support families by fostering trusting relationships, which form the foundation for mutually beneficial partnerships in long-term care. These partnerships are essential for helping families navigate challenges and transitions effectively [64, 65]. Service providers demonstrated flexibility by taking time to understand each family, engaging with them on their terms, remaining accessible, and responding promptly to concerns. This approach reflects a shift from people-centered to people-driven care, where families’ goals and needs actively shape service delivery, an essential condition for achieving integrated care [66].

To address changing care needs, service providers continuously adapted their practices, a process referred to as reframing services in response to specific situations [22]. These adaptations included offering situational support by deviating from standard protocols to meet families’ unique needs. Providers also reorganized services to reduce the number of care units involved, thereby streamlining coordination and improving continuity. Similar strategies have been documented in other empirical studies [21, 67], frequently described as goal-based rules that balance procedural compliance with necessary flexibility [24]. In this study, providers modified or bypassed procedures to integrate fragmented services that families found difficult to navigate, a challenge also reported in other child and family care contexts [58].

Service providers adapted their support for families through both planned and ad hoc measures, depending on care needs and evolving circumstances. This finding aligns with previous research [25], which describes short-term adaptations as strategies for managing immediate challenges, for example, skipping steps in follow-up protocols during consultations to address urgent concerns raised by families. Long-term adaptations involved engaging with parents early to address care needs from a life-course perspective, aiming to improve health outcomes and support the child’s development. These efforts also helped parents remain active participants in their child’s healthcare journey over time.

Providers demonstrated an understanding of the family as the unit of care by adapting their approach to complex treatment decisions. In specialist healthcare settings, they offered decision-making support to parents and, in some cases, postponed procedures, such as surgery, based on parents’ preferences and needs. These adaptations were often informed by providers’ prior experiences with families who found such decisions overwhelming due to uncertainty and responsibility. This reflects findings from earlier studies showing that parents vary in their preferences for involvement in care decisions: some wish to decide independently, others prefer shared decision-making, and some delegate decisions to providers [68, 69]. Therefore, decision-making practices should be tailored to the diverse and evolving needs of families to foster engagement and autonomy without adding stress or unwanted responsibility [15].

Service providers in this study adapted their practices to navigate divergent perspectives, both between families and other professionals, and within families themselves. These adaptations involved efforts to align perspectives, which research defines as the convergence of views, meanings, and practices to achieve shared outcomes [25]. Providers hold the responsibility of safeguarding the child’s best interests throughout service delivery. In some cases, they identified conflicting viewpoints through direct engagement with children and families.

Recent research emphasizes the importance of giving children a more central role in their healthcare and addressing the asymmetrical nature of care relationships, where providers and parents often dominate decision-making [10, 17, 70, 71]. This highlights the need to reframe children’s healthcare services toward a more child-centered approach, one that ensures the child’s voice is considered in care decisions and that their autonomy is respected.

### Strengths and limitations

A strength of this study was the combination of interviews and observations, which enabled the collection of in-depth information from multiple sources. The participation of service providers affiliated with a variety of different sectors, levels, and units offered diverse perspectives, allowing for a comprehensive exploration of the adaptations they enact to provide FCC to children with CP and their families. However, a limitation of the study was the exclusion of service providers such as general practitioners and additional medical specialists who also offer care to children with CP and their families. Further research should include a wider range of service providers to capture an even broader picture of the adaptations enacted. Additionally, children with CP and their families, who could provide feedback on whether service providers’ adaptations contribute to FCC, were not included in this study. Their perspectives on the care provided is addressed in Askeland et al. 2025 [27].

## Conclusion

Through the enactment of various adaptations to practice, service providers supported families by creating trusting relationships, ensuring early interventions and individualized care, and negotiating divergent perspectives. Service providers also adapted practice to aid care organization, thus facilitating collaboration and information management. The adaptations enacted show that service providers use their situational understanding to decide on and implement flexible approaches to FCC according to a given family’s care needs. This compensates for shortcomings in the service system to ensure coordinated and integrated care according to a family-centered approach. Further research from the family perspective is necessary to explore whether and how the adaptations described contribute to services that meet families’ care needs.

## Supplementary Information

Supplementary Material 1.

Supplementary Material 2.

Supplementary Material 3.

## Data Availability

The datasets are not publicly available due to confidentiality. However, they can be made available from the corresponding author upon reasonable request and pending ethics clearance from the Regional Committee for Medical and Health Research Ethics in Norway and the Norwegian Agency for Shared Services in Education and Research.

## Abbreviations

CP: Cerebral Palsy
FCC: Family centered care
